# Effects of Rhamnolipids on Growth Performance, Immune Function, and Cecal Microflora in Linnan Yellow Broilers Challenged with Lipopolysaccharides

**DOI:** 10.3390/antibiotics10080905

**Published:** 2021-07-24

**Authors:** Haoran Zhang, Xiaorong Yu, Qing Li, Guangtian Cao, Jie Feng, Yuanyuan Shen, Caimei Yang

**Affiliations:** 1 Key Laboratory of Applied Technology on Green-Eco-Healthy Animal Husbandry of Zhejiang Province, The Zhejiang Provincial Engineering Laboratory for Animal Health and Internet Technology, College of Animal Science and Technology, College of Animal Medicine, Zhejiang Agriculture & Forestry University, Hangzhou 311300, China; zhanganne49@163.com (H.Z.); xiaorongyu1998@168.com (X.Y.); 18806516157@163.com (Q.L.); Shenyuanyuan0708@168.com (Y.S.); 2 The Zhejiang Provincial Engineering Laboratory for Animal Health and Internet Technology, College of Animal Science and Technology, College of Animal Medicine, Zhejiang Agriculture & Forestry University, Hangzhou 311300, China; 3 College of Standardization, China Jiliang University, Hangzhou 310018, China; 15a1903025@cjlu.edu.cn; 4 College of Animal Sciences, Zhejiang University, Hangzhou 310058, China; fengj@zju.edu.cn

**Keywords:** rhamnolipid, lipopolysaccharide, microflora community, growth performance, broiler

## Abstract

This present study aimed to investigate the effects of rhamnolipids (RLS) on the growth performance, intestinal morphology, immune function, short-chain fatty acid content, and microflora community in broiler chickens challenged with lipopolysaccharides (LPS). A total of 450 broiler chickens were randomly allocated into three groups: basal diet with no supplement (NCO), basal diet with bacitracin (ANT), and basal diet with rhamnolipids (RLS). After 56 d of feeding, 20 healthy broilers were selected from each group, with half being intraperitoneally injected with lipopolysaccharides (LPS) and the other half with normal saline. Treatments with LPS were labelled LPS-NCO, LPS-ANT, and LPS-RLS, whereas treatments with normal saline were labelled NS-NCO, NS-ANT, and NS-RLS. LPS-challenged birds had lower jejunal villus height and higher crypt depth than unchallenged birds. LPS-RLS broilers had increased jejunal villus height and villus height/crypt depth ratio (V/C) but lower crypt depth than LPS-NCO. Dietary supplementation with RLS reduced the LPS-induced immunological stress. Compared with LPS-NCO, birds in LPS-RLS had lower concentrations of IL-1β, IL-6, and TNF-α. In LPS-challenged broilers, RLS and ANT increased the concentrations of IgA, IgM, and IgY compared with LPS-NCO. In LPS treatments, RLS enhanced the contents of acetic acid, butyrate, isobutyric acid, isovalerate, and valerate more than LPS-NCO birds. High-throughput sequencing indicated that RLS supplementation led to changes in the cecal microbial community of broilers. At the species level, *Clostridium-sp-Marseille-p3244* was more abundant in NS-RLS than in NS-NCO broilers. In summary, RLS improved the growth performance and relative abundance of cecal microbiota and reduced the LPS-induced immunological stress in broiler chickens.

## 1. Introduction

Antibiotics play an extremely important role in the livestock industry by improving the growth performance and efficiency of feed utilization, preventing disease, and reducing mortality and morbidity rates [1,2,3]. However, antibiotics used in feed might cause undesirable consequences to human health, pose a threat to food safety, and constitute an environmental problem [1,4]. To address this, National State Health Departments around the world have banned the use of antibiotics as growth promoters in animal diets [5]. Therefore, antibiotic alternatives are attracting increasing attention in the livestock industry.

Rhamnolipids (RLS), which are glycolipids generally isolated from *Pseudomonas aeruginosa*, have a number of distinctive abilities, such as tolerance to pH and temperature, low toxicity, and antimicrobial activity [6,7,8,9]. The hydrophilic–lipophilic balance value of RLS is 22–24, indicating that RLS have a strong solubilization effect [10,11]. In addition, RLS also have a strong oil–water emulsifying capacity [12]. Moreover, RLS not only exhibit excellent antibacterial, antifungal, and antiviral activities, but also have good anti-adhesive properties against pathogens [13,14]. In addition, RLS showed excellent antimicrobial properties against almost all tested bacteria, including *Enterobacter aerogenes*, *Staphylococcus epidermidis*, *Mycobacterium*, and *Arthrobacter* [15]. RLS may be added into foods as additives to stabilize the inflatable system, improve the taste and shelf life of starch food, change the rheological properties of flour, bond fat particles, and increase the consistency and taste of fat food. A report from the US Environmental Protection Agency (USEPA, 2004) evaluated the biological safety of rhamnolipids as an additive to poultry feed and exempted the toxicity testing.

Lipopolysaccharides (LPS), the essential outer membrane components of almost all Gram-negative bacteria, are known to stimulate immune responses to stresses in the host [16]. In the body, LPS might cause natural or innate immune responses, leading to elevation of inflammatory cytokines and a decrease in the immune function [17,18]. Previous studies extensively explored the implementation of RLS in the agricultural environment, as well as in petroleum and food processing [19,20,21]. However, few studies have evaluated whether RLS could be used as alternatives to antibiotic growth promoters in livestock and poultry. The current study was conducted to determine the effects of feeding RLS on the growth performance, intestinal morphology, immune function, short-chain fatty acids (SCFAs), and cecal microbiota in broiler chickens challenged with LPS.

## 2. Results

### 2.1. Growth Performance

The data are presented in Table 1. Broilers supplemented with RLS had an improved body weight (BW) and average daily gain (ADG) compared with the control treatment (*p* < 0.05). Moreover, we did not observe any major differences in body weight (BW), average daily gain (ADG), and F:G between RLS- and ANT-treated broilers.

### 2.2. Morphological Analysis of Jejunum

**Figure 1:**
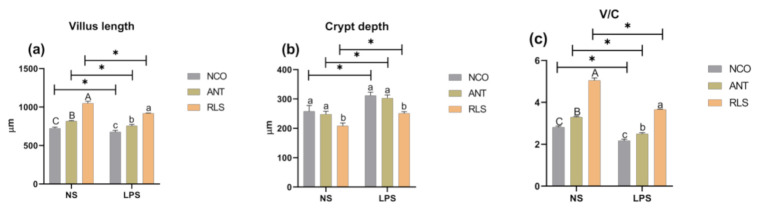
Effects of RLS on jejunum morphology in broilers challenged with LPS. (**a**) Effect of RLS on villus length. (**b**) Effect of RLS on crypt depth. (**c**) Effect of RLS on villus height/crypt depth ratio (V/C). NS-NCO represents control broilers treated with normal saline. NS-ANT represents broilers treated with the antibiotic; NS-RLS represents broilers treated with rhamnolipids; LPS-NCO represents control broilers challenged with LPS; LPS-ANT represents broilers treated with the antibiotic and challenged with LPS; LPS-RLS represents broilers treated with rhamnolipids and challenged with LPS; * and different superscript letters indicate significant differences (*p* < 0.05), majuscule present NS-groups and lowercase present LPS-groups, *n* = 6.

We found that the jejunal villus length and villus height/crypt depth ratio (V/C) of NS-RLS- and NS-ANT-treated broilers were prominently increased relative to those of control (Figure 1). Moreover, we noted that NS-RLS broilers had lower crypt depth than NS-NCO broilers (*p* < 0.05), while injection of LPS was found to significantly decrease the villus height and increase the V/C compared with the NS-NCO treatment. In addition, broilers in the LPS-RLS and LPS-ANT groups were demonstrated to have higher villus height and V/C relative to those in the LPS-NCO group (*p* < 0.05).

### 2.3. Immunoglobulins

**Figure 2:**
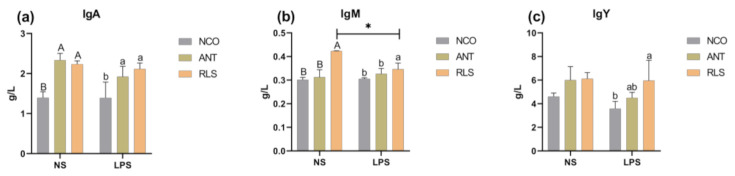
Effects of RLS on immunoglobulins in broilers challenged with LPS. (**a**) Effect of RLS on IgA. (**b**) Effect of RLS on IgM. (**c**) Effect of RLS on IgY. NS-NCO represents control broilers treated with normal saline; NS-ANT represents broilers treated with the antibiotic; NS-RLS represents broilers treated with rhamnolipids; LPS-NCO represents control broilers challenged with LPS; LPS-ANT represents broilers treated with the antibiotic and challenged with LPS; LPS-RLS represents broilers treated with rhamnolipids and challenged with LPS; * and different superscript letters indicate significant differences (*p* < 0.05), majuscule present NS-groups and lowercase present LPS-groups, *n* = 6.

We observed that in saline-treated groups, birds supplemented with RLS had higher levels of IgA and IgM compared with those in the control group (*p* < 0.05; Figure 2). In LPS-stimulated birds, both RLS and ANT treatments were shown to remarkably increase the concentration of IgA, IgM, and IgY compared with those of the control-group birds (*p* < 0.05).

### 2.4. Serum Inflammatory Factors

**Figure 3:**
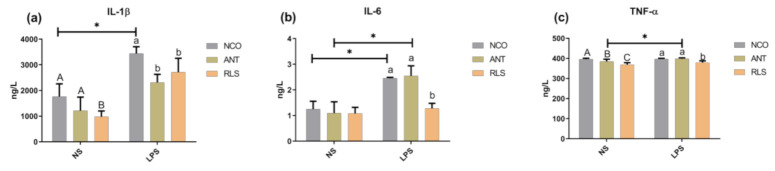
Effects of RLS on serum inflammatory factors in broilers challenged with LPS. (**a**) Effect of RLS on IL-1β. (**b**) Effect of RLS on IL-6. (**c**) Effect of RLS on TNF-α. NS-NCO represents control broilers treated with normal saline; NS-ANT represents broilers treated with the antibiotic; NS-RLS represents broilers treated with rhamnolipids; LPS-NCO represents control broilers challenged with LPS; LPS-ANT represents broilers treated with the antibiotic and challenged with LPS; LPS-RLS represents broilers treated with rhamnolipids and challenged with LPS; * and different superscript letters indicate significant differences (*p* < 0.05), majuscule present NS-groups and lowercase present LPS-groups, *n* = 6.

We found that birds in the NS-RLS- and NS-ANT-treated groups had lower concentrations of IL-1β, IL-6, and TNF-α compared with those in control birds (*p* < 0.05; Figure 3), whereas the levels of IL-1β and IL-6 were shown to be significantly increased in LPS-challenged birds compared with those of the saline-treated ones (*p* < 0.05). We further noted that compared with LPS-CON, LPS-RLS birds had lower levels of IL-1β, IL-6, and TNF-α (*p* < 0.05).

### 2.5. Short-Chain Fatty Acids in Colon Morphology

**Figure 4:**
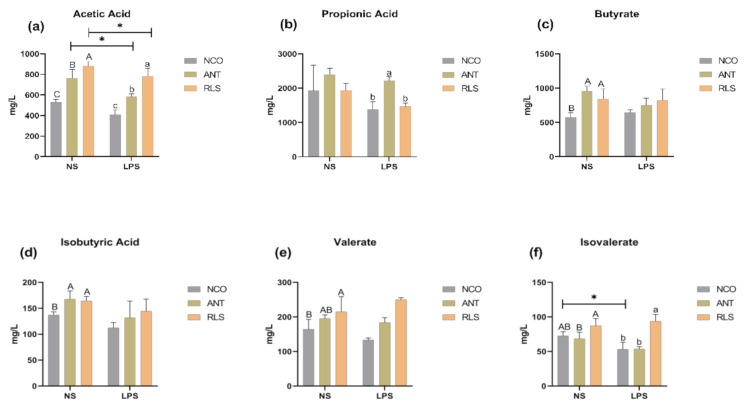
Effects of RLS on SCFAs in colon morphology in broilers challenged with LPS. (**a**) Effect of RLS on acetic acids. (**b**) Effect of RLS on prophionic acid. (**c**) Effect of RLS on butyrate. (**d**) Effect of RLS on isonutyric acid. (**e**) Effect of RLS on valerate. (**f**) Effect of RLS on isovalerate. NS-NCO represents control broilers treated with normal saline; NS-ANT represents broilers treated with the antibiotic; NS-RLS represents broilers treated with rhamnolipids; LPS-NCO represents control broilers challenged with LPS; LPS-ANT represents broilers treated with the antibiotic and challenged with LPS; LPS-RLS represents broilers treated with rhamnolipids and challenged with LPS; * and different superscript letters indicate significant differences (*p* < 0.05), majuscule present NS-groups and lowercase present LPS-groups, *n* = 6.

Compared with birds in the NS-NCO group, birds fed with RLS and ANT were demonstrated to have higher levels of acetic acid, butyrate, isobutyric acid, and valerate (*p* < 0.05; Figure 4a,c–e). Birds in the LPS-ANT group had evidence of higher levels of acetic acid and valerate (*p* < 0.05; Figure 4a,f). Birds in the LPS-RLS group were further shown to have higher concentrations of acetic acid, valerate, and isovalerate compared with those in the LPS-NCO group (*p* < 0.05; Figure 4a,e,f).

### 2.6. Summary of Microbial Community in Caecum Contents of Broilers

**Figure 5:**
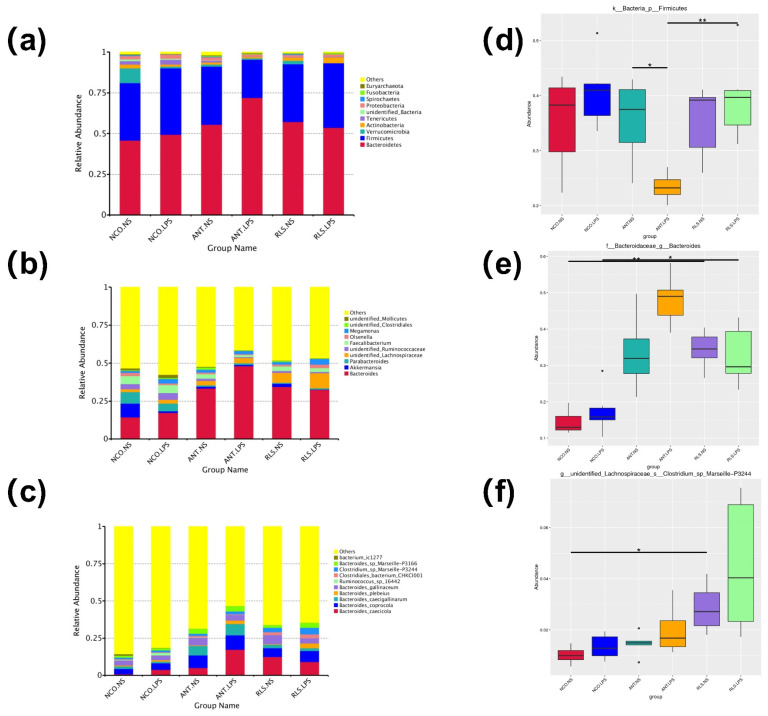
Effects of RLS on microbial community in caecum contents in broilers challenged with LPS. (**a**–**c**) The top 10 taxa by relative abundance within the microflora community ((**a**): phylum; (**b**): genus; (**c**): species). (**d**–**f**) Species with significant inter-group differences (**d**): phylum; (**e**): genus; (**f**): genus. NS-NCO represents control broilers treated with normal saline; NS-ANT represents broilers treated with the antibiotic; NS-RLS represents broilers treated with rhamnolipids; LPS-NCO represents control broilers challenged with LPS; LPS-ANT represents broilers treated with the antibiotic and challenged with LPS; LPS-RLS represents broilers treated with rhamnolipids and challenged with LPS; * and different superscript letters indicate significant differences (*p* < 0.05), ** represents very significant differences (*p* < 0.01). *n* = 6.

We detected that the three major phyla were *Firmicutes, Bacteroidetes, Actinobacteria*, and *Tenericutes* in the RLS groups (Figure 5a). The relative abundance of *Firmicutes* in the LPS-RLS group was obviously higher than that in the LPS-ANT group (*p* < 0.05; Figure 5d). At the genus level, *Bacteroides, Parabacteroides, Akkermansia, Faecalibacterium, Megamonas,* and *Olsenella* were the predominate genera identified in all samples (Figure 5b). We found that the relative abundance of *Bacteroides* in the NS-RLS group was notably higher compared to the NS-NCO group (*p* < 0.05; Figure 5e). Furthermore, the relative abundance of *Bacteroides* was also demonstrated to be significantly higher in the LPS-RLS group than in the LPS-NCO group (*p* < 0.05; Figure 5e). At the species level, we found that *Bacteroides caecicola, B. coprocola, B. caecigallinarum, B. plebeius, B. gallinaceum, Ruminococcus-sp-16442, Clostridiales-bacterium-chkcl001, Clostridium-sp-Marseille-p3244*, *Bacteroides-sp-Marseille-p3166,* and *Bacterium-ic1277* were the dominant species in all groups (Figure 5c). Interestingly, *Clostridium-sp-Marseille-p3244* was shown to be more abundant in the NS-RSL compared with the NS-NCO group (Figure 5f).

## 3. Discussion

Antibiotic substitutes have received increasing attention in recent years. As RLS have several desirable characteristics, including the regulation of the immune system, and antibacterial and bacteriostatic abilities, they have been proposed as potential antibiotic substitutes [22,23,24]. Previous studies in our own laboratories suggested that broilers fed a diet supplemented with 1000 mg/kg RLS exhibited an improved body weight [25]. However, there have only been few studies on the current status of broilers fed diets supplemented with RLS. In our study, RLS significantly increased the BW and ADG indexes compared with those of the control-group birds. However, additional research is required to probe into the effect of RLS on the growth performance of broiler chickens.

The height of villus and depth of crypt are known to directly reflect the gut function and health, and are common indicators for estimating intestinal integrity. [26]. The villus height relates to the surface area for nutrient absorption and crypt depth reflects the formation rate of villus epithelial cells. The shallow crypt indicates whether the rate of cell maturation is increased and secretory function is enhanced, which are associated with nutrient digestion and absorption capacity [27]. An increase in the length of the intestinal villus could strengthen the contact between the intestine and nutrients, thus improving digestion and absorption [28]. However, injection with LPS increased the ileum crypt depth and decreased the ratio of villus height to crypt depth in broilers [29]. However, few studies have demonstrated the impact of RLS on jejunal morphology. Our results showed that broilers in the RLS groups exhibited increased values of villus length and villus height/crypt depth (V/C). Furthermore, in the LPS-stimulated groups, RLS was demonstrated to be superior in protecting the intestinal development and promoting the maintenance of normal intestinal function compared with ANT.

Owing to their important roles in immune functions, the concentration of immunoglobulins can be used as a parameter reflecting the immune status of animals. Birds are known to produce three different antibody isotypes: IgM, IgA, and IgY [30]. IgY, which is the functional immunoglobulin and major circulating antibody found in birds, plays a similar biological role to that of IgG in mammals [31,32]. In our study, stimulation of birds with LPS decreased the level of IgY, whereas the addition of RLS maintained the level of IgY. We also found that compared with antibiotics, adding RLS significantly increased the level of IgM in unchallenged birds. In human mononuclear cells, a heat-stable extracellular toxin with similar structure to RLS stimulated immune cells to induce the release of TNF-α [33]. However, RLS decreased the level of TNF-α in human MNCs compared with rough mutant lipopolysaccharide (LPS) [34]. RLS activated innate immune responses in human epithelial cells, especially inducing the generation and release of pro-inflammatory cytokines, such as IL-8 [35]. In our study, the addition of RLS after stimulation with LPS significantly decreased the level of IL-1β, IL-6, and TNF-α. 

Short-chain fatty acids (SCFAs), the main metabolite products of the fermentation of dietary fibers by anaerobic intestinal microbiota, are known to play a role in improving intestinal disorders via the provision of the energy source for the growth of anaerobic bacteria and the inhibition of the growth of harmful bacteria [36,37]. Adding RLS into dewatered oily sludge led to the acceleration of its hydrolysis and fermentation, resulting in turn in an increase in SCFAs [38]. During the process of hydrolysis and acidification of waste-activated sludge (WAS), RLS greatly reduced the surface tension of sludge, resulting in the stimulation of the hydrolysis rate of WAS and the enhancement of the production of SCFAs. Acetic, propionic, and isovaleric acids were the three main products at any pH value when the accumulation of SCFAs reached the highest values [39,40]. In our results, compared with birds injected with normal saline, birds treated with RLS and ANT had higher levels of acetic acid, butyrate, isobutyric acid, and valerate. Likewise, the RLS groups could relieve the LPS-induced decrease in the levels of acetic acid, propionic acid, isobutyric acid, valerate, and isovalerate. Treatment of LPS-stimulated birds with RLS and ANT induced an increase of acetic acid, isobutyric acid, and valerate compared with those in the control group.

A wide variety of intestinal bacteria are known to form a large and complex micro-ecological system that evolves with the host, and is directly involved in many aspects, such as digestion, nutrient absorption, energy supply, fat metabolism, immune regulation, and disease resistance [41,42,43,44]. Cecal microbiota can provide energy in the form of amino acids and SCFAs, which benefit the host through changes in intestinal morphology, including modifications of the villus height and crypt depth [45,46].

In broilers, cecal communities have been shown to be dominated by *Firmicutes* and *Bacteroides* at the phylum level [47]. There are very significant correlations between the relative amounts of *Bacteroides* and nutrient digestibility values in broiler ileal samples [48]. *Bacteroides* are known to be major producers of branched-chain amino acids, acetic acid, and butyric acid [49,50]. In addition, the most abundant phyla involved in the fermentation of glucose were *Firmicutes*, *Bacteroidetes*, and *Proteobacteria,* of which especially *Firmicutes* (genus *Clostridium*) were connected to the highest production of SCFAs [51]. *Firmicutes* are known to produce butyrate and other SCFAs to maintain intestinal health by suppressing inflammation and providing energy to enterocytes [52,53]. According to our results, C*lostridium-sp-Marseille-p3244* belonging to *Lachnospiraceae* was a butyrate-producing bacteria. *Lachnospiraceae* are in the position to produce butyrate by polysaccharides fermentation or by converting other SCFAs to butyrate [54,55] According our results, the concentrations of C*lostridium-sp-Marseille-p3244* and levels of butyrate showed the same trends. In the present study, we observed that *Firmicutes* and *Bacteroidetes* were the primary bacteria at the level of phylum in the RLS groups. The relative abundance of *Firmicutes* in the LPS-RLS group was obviously higher than that in the ANT-LPS group. At the genus level, our results showed that RLS increased the diversity of cecal microbiota, accompanied with increases in the numbers of *Bacteroides*.

## 4. Materials and Methods

### 4.1. Animals, Treatment, and Designation

This study was managed by the proposal on the protection and utilization of laboratory animals of the Institutional Animal Care and Use Committee of Zhejiang Agricultural and Forestry University. The agreement was confirmed by the Ethics Committee of Zhejiang Agricultural and Forestry University, Hangzhou, China (SYXKzhe 2019-054).

A total of 450 1-d-old Linnan yellow broiler chickens were randomly assigned to three treatments, with 10 pens in each treatment and 15 broilers per pen. A basic diet designed to meet the nutritional requirements recommended by NRC 1998 without antibiotics was used (Table 2). Depending on the group to which they belonged, birds were fed as follows: basal diet with no supplement (NCO), basal diet with 30 mg/kg bacitracin (ANT), basal diet with 1000 mg/kg rhamnolipids (RLS). Broilers were housed in electrically heated cages, and provided with water and the relevant diet ad libitum for 56 d. The broilers and poultry feed were weighed on day 56 after feed deprivation for 2 h.

**Figure 6:**
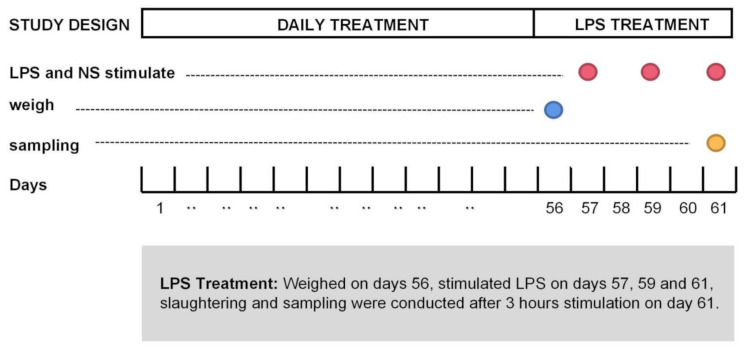
Design of LPS treatment. The blue circle represents the date of weighting, red circles represent the date of stimulated by rhamnolipids (LPS) or normal saline (NS) and the yellow circle represents the date of sampling.

On day 57, 20 healthy broilers of similar body weight selected from each group (60 broilers in total) were divided into two treatments. Briefly, 10 broilers were intraperitoneally injected with LPS at a dosage of 500 µg/kg body weight on days 57, 59, and 61, whereas the remaining 10 broilers were injected with normal saline on the same days (Figure 6). Broilers were humanely sacrificed prior to tissue collection. Treatments with LPS were labelled LPS-NCO, LPS-ANT, and LPS-RLS, respectively, whereas treatments with normal saline were labelled NS-NCO, NS-ANT, and NS-RLS.

Rhamnolipids were absorbed onto silica to form a uniformly dispersed powdery solid and were provided by Zhejiang Vegamax Biological Technology Co. Ltd. (Anji, Zhejiang, China).

### 4.2. Sample Collection

At 3 h after the third LPS challenge, all 60 broiler chickens were subjected to blood sampling and collection of the jejunal segment and cecal contents. Blood samples were taken from the front cavity vein and centrifuged (3000× *g*, 10 min) at 4 °C. Broilers were humanely sacrificed after blood collection by cervical dislocation. The separated serum was stored in the Eppendorf tubes at −80 °C until further analysis. Segments (approximately 1 cm) were removed from jejunum, which is located 5 cm behind the duodenum, after being gently washed with sterilized physiological saline solution. They were fixed in 4% formaldehyde (Aladdin, Shanghai, China) and stored at 4 °C for morphological evaluation. Meanwhile, cecal contents were immediately squeezed into a sterile tube and stored at −80 °C until analysis.

### 4.3. Growth Performance

The growth performance was evaluated by body weight (BW, g), average daily gain (ADG) and the ratio of feed to gain (F/G). On day 56, we removed feed for 2 h and recorded the remaining feed per pan, then measured the BW and feed intake of the broilers to calculate ADG and F/G.

### 4.4. Serum Immunologic Indexes

The concentration of the immunoglobulin A (IgA), IgM, IgY, interleukin 1β (IL-1β), interleukin 6 (IL-6), and tumor necrosis factor-α (TNF-α) serum proteins were measured by commercial kits (Huamei Biological Engineering Research Institute, Wuhan, China). These assays were conducted in accordance with the manufacturer’s instructions.

### 4.5. Morphological Analysis of Jejunum

The 0.5 cm jejunum segments were fixed with 10% formaldehyde (Aladdin, Shanghai, China) solution for a minimum of 24 h, dehydrated, embedded in paraffin (4 μm), and stained with hematoxylin-eosin. Sections were mounted for observation and photographed under a fluorescence microscope (Eclipse Ci equipped with a DS-FI2 camera, Nikon, Japan). The height of villus and crypt of depth were measured at 40× magnification on 10 visual fields for each sample. Height of villus was determined from the top of villus to the connection of villus and crypt, crypt depth was defined as the depth of emboly between nearby villi. The villus height and crypt depth ratios were calculated from these measured data.

### 4.6. Analysis of Short-Chain Fatty Acids

Short-chain fatty acid concentration in cecal was measured using the gas chromatography procedure. Briefly, 1 g cecal content was mixed with 1 mL dd H_2_O. After mixing and centrifugation (4 °C, 1200× *g*), the supernatant was removed. The supernatant was transferred and mixed with 25% metaphosphoric acid (*w/v*, 5:1; AR, Aladdin, Shanghai, China). After 30 min at 4 °C, the tubes were centrifuged again. Analyses were performed using Agilent 7890B GC Network System (Agilent Technologies, Wilmington, DE, USA) equipped with a 30 m × 0.25 mm × 0.25 µm column (HP-FFAP, Agilent Technologies) for flame ionization [56].

### 4.7. 16S rRNA Sequencing of Cecal Microflora

A total of 36 cecal content samples were used for analysis of the intestinal flora. The V4 region of the 16S rRNA gene was explored using an Illumina-Hiseq platform (Novogene Bioinformatics Technology Co. Ltd.; Beijing, China). Shortly, using the Quantitative Insights into Microbial Ecology (QIIME, http://qiime.org/, accessed on 1 December 2019) and Uparse (https://drive5.com/uparse/, accessed on 1 June 2020) software, we gained 97% similarity between taxa and Ribosomal Database Project classifiers. The operational taxonomic unit (OTU) clustering and species classification analysis was based on valid data. Species annotations were made for each clustered OTU sequence, and the relative species information and species-based abundance distribution were obtained.

### 4.8. Statistical Analysis

Statistical analysis was performed using the Prism 8.0 software (GraphPad Software Inc., San Diego, CA, USA) and SPSS 26.0 software (IBM Corp, New York, NY, USA). One-way ANOVA analyzed valid data and *p* < 0.05 was considered statistically significant.

## 5. Conclusions

Dietary supplementation with RLS improved the growth performance and relieved the stress elicited by LPS in broiler chickens, and raised the relative abundance of cecal microbiota. Moreover, RLS benefited the intestinal morphology, regulated the host immune function, and enhanced the level of SCFAs in colon contents.

## Figures and Tables

**Table 1:** Effects of RLS on growth performance in broilers.

Items	Treatments			SEM	*p*-Value
	NCO	ANT	RLS		
Body weight, g					
1 d	35.44	35.31	35.25	0.16	0.89
56 d	1477.55 ^b^	1523.60 ^a,b^	1575.11 ^a^	12.99	0.01
Average daily gain, (g/d)					
1–56 d	26.22 ^b^	27.06 ^a,b^	28.00 ^a^	0.24	0.01
F:G, (g/g)					
1–56 d	2.37	2.41	2.46	0.02	0.68

Values in the same line with no letter or the same superscript letter show no significant difference (*p* > 0.05), whereas values with different superscript letters indicate significant difference (*p* < 0.05). NCO-, ANT-, RLS- represent broilers fed basal diet, broilers fed antibiotic, and broilers fed rhamnolipids, respectively; *n* = 6 per treatment.

**Table 2:** Composition and nutrition levels of basal diet.

Items	1 to 28 Days of Age	29 to 56 Days of Age
Ingredients	/	/
Corn	53	53
Soybean meal	24.5	16
Extruded soybean	5	3
DDGS	8	8
Rice bran	/	8
Corn gluten	/	2
Soybean oil	1.7	4.5
Limestone	1.3	1.5
Fermented soybean meal	2.5	/
Premix1	4	4
Total	100.00	100.00
Nutrient levels	/	/
Crude protein	20.3	17.2
ME (MJ/kg)	2916	3090
Crude fat	5.5	8.6
Lysine	1.19	0.96
Methionine	0.54	0.44
Methionine + Cysteine	0.89	0.74
Threonine	0.86	0.71
Tryptophan	0.23	0.20
Calcium	0.87	0.73
Total Phosphorus	0.60	0.57

Concentrate mixture provided the following per kilogram of complete diet: 1500 IU of vitamin A; 200 IU of vitamin D3; 10 IU of vitamin E; 35 g of vitamin K; 1.5 mg of vitamin B1; 3.5 mg of vitamin B2; 3 mg of vitamin B6; 10 μg of vitamin B12; 10 mg of pantothenic acid; 30 mg of nicotinic acid; 0.15 mg of biotin; 1000 mg of choline chloride; 8 mg of copper; 60 mg of manganese; 80 mg of iron; 40 mg of zinc; 0.18 mg of iodine; 0.15 mg of selenium.

## Data Availability

The data presented in this study are available on request from the corresponding author.
